# Multiple Paternity in Polyandrous Barn Owls (*Tyto alba*)

**DOI:** 10.1371/journal.pone.0080112

**Published:** 2013-11-11

**Authors:** Isabelle Henry, Sylvain Antoniazza, Sylvain Dubey, Céline Simon, Céline Waldvogel, Reto Burri, Alexandre Roulin

**Affiliations:** Department of Ecology and Evolution, Biophore Building, University of Lausanne, Lausanne, Switzerland; Institute of Ecology, Germany

## Abstract

In polyandrous species females produce successive clutches with several males. Female barn owls (*Tyto alba*) often desert their offspring and mate to produce a 2^nd^ annual brood with a second male. We tested whether copulating during chick rearing at the 1^st^ annual brood increases the male's likelihood to obtain paternity at the 2^nd^ annual breeding attempt of his female mate in case she deserts their brood to produce a second brood with a different male. Using molecular paternity analyses we found that 2 out of 26 (8%) second annual broods of deserting females contained in total 6 extra-pair young out of 15 nestlings. These young were all sired by the male with whom the female had produced the 1^st^ annual brood. In contrast, none of the 49 1^st^ annual breeding attempts (219 offspring) and of the 20 2^nd^ annual breeding attempts (93 offspring) of non-deserting females contained extra-pair young. We suggest that female desertion can select male counter-strategies to increase paternity and hence individual fitness. Alternatively, females may copulate with the 1^st^ male to derive genetic benefits, since he is usually of higher quality than the 2^nd^ male which is commonly a yearling individual.

## Introduction

In most species with parental care, the investment of the two parents is unequal, with the mother usually contributing more than the father to parental duties. Differences in parental investment are rooted in the initial investment in gametes, with females producing larger eggs than the males' sperm cells (i.e. anisogamy). This asymmetric situation is the source of conflict between the sexes over their investment [1]–[3]. The most extreme form of conflict is when one parent decides to abandon its progeny and its partner. The decision to care for or desert offspring in search of new mating opportunities has long been recognized as a source of conflict between genders. Whether a parent should stay or leave depends on the probability of finding a new mate and whether the presence of both parents is required to successfully raise progeny [4], [5]. When the presence e.g. of the father is not necessary to ensure offspring survival, males desert soon after copulation, because caring for offspring entails the cost of lost mating opportunities [6]. Because males can more quickly replenish their relatively cheaper sperm than females can replace their costly eggs, males are usually predicted to invest more heavily in traits that increase mating success rather than investment in offspring care. However, if adult sex ratio is evenly balanced, males who are ready to mate may be unable to find a currently fertile female partner, depending on the degree of breeding seasonality of the species. Indeed, on average, males will have to wait for females to become sexually available. Hence, it may be advantageous for them, in term of fitness prospect, to take care of their offspring (instead of deserting), during periods when females are not available for mating.

Although in most of the species with parental care, the female is the primary caregiver, there are notable exceptions. For example, in birds, many species are monogamous with males participating in parental care (about 90% of bird species, [7]). With the exception of a few polygamous species where males do not participate in parental care but compete to secure more than one female, monogamous males can trade-off their investment in offspring rearing against the search of extra-pair copulations by decreasing their level of care [8]. The situation can be even more complex in species that produce several broods per year. Indeed, the temptation is high to abandon the brood to its mate in order to start a second breeding attempt with a mate free of parental duties. This situation is particularly interesting because it is not evident at first sight which of male or female should desert its brood. Depending on the costs and benefits of desertion, one of the two sexes may be more likely to desert. This decision will depend on sex ratio of available mates and on which parent is most useful to pursue offspring rearing, as well as resources availability (e.g. [9], [10]). If more males than females are not breeding and hence available to produce a brood late in the season, females may be more tempted to desert their brood and start a new one [11], as has been shown for example in the rock sparrow (*Petronia petronia*) [12]. Furthermore, if the fitness of offspring is more sensitive to male than female care, males may be less tempted to desert than females. This is the case in the barn owl (*Tyto alba*) and Tengmalm's owl (*Aegolius funereus*) in which the presence of the mother is not mandatory to raise the offspring between the period when they can consume food without maternal help and independence [13]–[15]. Females frequently desert their mate and offspring halfway through the rearing period to produce a 2^nd^ annual brood with another male. The first male may thus frequently copulate during offspring rearing in case his female deserts to produce a 2^nd^ annual brood with another male. Although copulation frequency is maximal during egg laying (on average more than one copulation per hour), barn owls can continue to copulate during the entire rearing period (but at a lower rate) particularly when owls are planning to produce a 2^nd^ annual brood [16], [17]. This behaviour may increase the likelihood that the male sires some of the offspring of the female's 2^nd^ brood in case she suddenly deserts him [18]. Although in raptors and owls extra-pair paternity is usually very low (less than 1% of the broods contain extra-pair young, i.e. young sired by another male than the one that feeds them [19]), we predict that 2^nd^ annual broods of deserting females entail a number of young sired by the male with which these females produced the 1^st^ annual brood. We tested this prediction with paternity analyses of the 1^st^ and 2^nd^ annual broods of female barn owls that produced these two successive broods with a single or with two different males.

## Materials and Methods

### Study organism

In a Swiss population of barn owls, about 10% of the breeding females produce two broods per year (this figure can be much higher in other countries, e.g. [20]) and we already showed that 43% of the double-brooded females abandon their offspring before the end of the rearing period to re-mate at a distance of 1.5 to 10 km and start a 2^nd^ annual breeding attempt with a new mate [13]. Whereas the reproductive success of faithful and divorced females did not differ in terms of number or condition of offspring, the 2^nd^ annual clutch of deserting females was laid and therefore hatched on average two weeks earlier than the 2^nd^ clutch of faithful females [13]. Unfortunately, it is unknown whether females copulate more frequently with the first or second male.

### General method

We conducted the study between 1996 and 2011 in a wild population of Barn Owls nesting in nest-boxes in the region of Payerne, Switzerland (46°49′N, 6°57′E, altitude 490 m). The area covers 480 km^2^ (see [21] for a scaled map of the study area). We checked nest-boxes regularly throughout the breeding season (April-October) to record 1^st^ and 2^nd^ annual broods, ring all birds with a unique number and collect blood samples in the two parents and their offspring to run paternity analyses. We obtained legal authorizations to collect blood samples from the “Service vétérinaire du canton de Vaud” and to ring owls from the Swiss Ornithological Institute of Sempach.

### Microsatellite genotyping

Blood samples were collected from the brachial vein using Heparin-coated tubes, immediately placed on dry ice and stored at −20°C. Genomic DNA was later extracted from blood using the DNeasy Tissue Kit (QIAGEN). Polymerase chain reactions (PCR) were performed in a final volume of 8 µL containing 1.4 µL H_2_O, 2.5 µL QIAGEN Multiplex PCR Master Mix, and 1.1 µl of all 6 pairs of primers premixed [between 0.08 and 0.45 µl of each fluorescent-labelled forward primer (2× 6-FAM, 2× HEX and 2× NED) and non-labeled reverse primer (primer concentration for all primers could be provided upon request)]. Twelve nanograms DNA were used as a template. PCR conditions included an initial denaturation step at 95°C for 15 min, 34 cycles of denaturation at 94°C for 30 s, primer annealing at 57°C for 90 s, and primer extension at 72°C for 1 min. A final step at 60°C for 30 min was used to complete primer extension. Fragment analysis was run on an ABI 3100 automated sequencer (Applied Biosystems), and allele sizes were assigned using genemapper 3.7 software (Applied Biosystems). Six microsatellite loci (Ta204, Ta206, Ta216, Ta310, Ta413 and Ta414; [22]) were analyzed. We used two different polymers to genotype individuals collected between 1996 and 2009 and between 2010 and 2011, and therefore analyzed the two datasets separately.

Between 1996 and 2009, we obtained complete pedigree and genetic data for 343 nestlings from 70 broods produced by 34 different breeding females and 50 different breeding males. We also included the genotype of 77 additional adults that bred during this period in the dataset (36 females and 41 males). The dataset in 2010 and 2011 contained 112 nestlings from 20 broods produced by 9 different females and 11 different males. The genotypes of 68 additional breeding adults were also included (32 females and 36 males). In total we genotyped 219 nestlings from 49 1^st^ annual broods (from 44 different females and 45 males), 93 nestlings from 20 2^nd^ annual broods in the case the mother bred with the same male as at the 1^st^ annual brood (produced by 17 different females and 17 different males) and 143 nestlings from 26 2^nd^ annual broods in the case the mother bred with another male than the one at the 1^st^ annual brood (produced by 26 different females and 26 different males). We considered only broods for which we captured and genotyped both parents.

### Linkage between markers

We tested for genotypic disequilibrium between loci and deviation from Hardy–Weinberg equilibrium was tested to check for the presence of allelic dropouts, null alleles, substructure and inbreeding. Deviations from random mating within populations (F_IS_) per locus and sample were computed with a bootstrap procedure of 120 randomizations. F_IS_ values were not significantly different from zero (Table 1) and no linkage disequilibrium between pairs of loci was detected (all *P*-values < adjusted 5% level). All summary statistics and tests were computed using FSTAT Version 2.9.3 [23]. Finally, significance values were corrected for multiple tests using the sequential Bonferroni method [24].

**Table 1:** Summary statistics in the adult barn owl population for all six microsatellite loci.

Locus	Dataset	No. Alleles	Allele size range (bp)	HO	HE	H-W	Null allele frequency	FIS	Larger FIS	Smaller FIS
Ta204	1996–2009	8	115–132	0.702	0.777	NS	0.0541	0.097	NS	NS
Ta206	1996–2009	12	262–289	0.849	0.852	NS	−0.0006	0.004	NS	NS
Ta216	1996–2009	16	181–235	0.72	0.751	NS	0.0194	0.041	NS	NS
Ta310	1996–2009	7	268–296	0.739	0.666	NS	−0.0594	−0.111	NS	NS
Ta413	1996–2009	15	170–225	0.9	0.894	NS	−0.0047	−0.007	NS	NS
Ta414	1996–2009	45	236–437	0.957	0.958	ND	−0.0008	0.002	NS	NS
Ta204	2010–2011	9	116–133	0.727	0.767	NS	0.0241	0.052	NS	NS
Ta206	2010–2011	13	266–291	0.943	0.872	ND	−0.0442	−0.082	NS	NS
Ta216	2010–2011	15	176–225	0.795	0.771	NS	−0.0213	−0.032	NS	NS
Ta310	2010–2011	8	272–299	0.69	0.713	NS	0.0091	0.033	NS	NS
Ta413	2010–2011	17	172–238	0.943	0.91	ND	−0.0218	−0.037	NS	NS
Ta414	2010–2011	43	241–433	0.989	0.961	ND	−0.0174	−0.029	NS	NS

Bp is for base pairs, NS for not significant after Bonferroni correction, ND for not tested, H-W for Hardy-Weinberg equilibrium, and HO and HE for observed and expected heterozygosity respectively.

### Parentage analysis

We used the software Cervus 3.0 [25] to determine parentage using a likelihood-based approach. To assess the confidence of the parentage assignment, we first estimated the allele frequencies in our population using the genetic data obtained from 70 breeding females and 91 breeding males captured between 1996 and 2009, and genetic data from 41 breeding females and 47 breeding males captured in 2010 and 2011. We then performed simulation of parentage analysis with 10′000 simulated offspring genotypes, assuming a 95% probability of sampling for the candidate mother and father, 99.5% of loci typed and allowing for 1% of loci mistyped. Because these simulations are sensitive to the number of candidate parents, we used the total number of breeding adults within each dataset as the number of candidate mothers and fathers used in the simulations. The probability of not excluding an unrelated father of a given offspring was in each case < 4*10^−6^, therefore ensuring a very high probability of correct parentage assignment.

Each nestling was tested in Cervus against the male mates of the mother at her 1^st^ and 2^nd^ annual clutches. Since no case of intraspecific brood parasitism was ever detected, we considered in our analyses that the mother was known with certainty. The software conducts a comparison between an offspring–mother pair and all the potential fathers in the dataset (in our case two per nestling), and calculates a LOD score (i.e. the logarithm of the likelihood ratio) for every potential father. The difference between the LOD scores of the male with the highest value and the second male is the Δ-criterion (Δ LOD) [26]. Δ LOD is compared with the critical Δ values calculated after a simulation and provided with a statistical confidence level. The levels of confidence for parentage assignment were 80% (relaxed) and 95% (strict) as used in the default settings.

## Results

All of the 455 assigned offspring had a level of confidence for parentage pair assignment higher than 95% and zero mismatches with their assigned biological father. However, concerning three nestlings of 1^st^ annual broods, the discrimination power between the two potential fathers was very low, due to similarities in their genotypes (i.e. the nestlings had zero mismatches with both of their potential fathers). We therefore excluded them from the results.

We detected extra-pair nestlings in two 2^nd^ broods produced by deserting females who produced their 2^nd^ annual brood with another male than the one with whom they produced the 1^st^ annual brood. Paternity analyses showed that the males at the 1^st^ breeding attempt sired the six extra-pair young (3 out of 8 young in one brood and 3 out of 7 young in the other brood). The two nests of these deserting females were located at 980 m and 1500 m distance, respectively, and at the first nest the offspring were aged 44 and 40 days at the time when their mother laid her first egg at the 2^nd^ annual clutch with a new mate. In comparison, the mean distance between the two nests of deserting females, for which all the offspring had been sired by the social male, was 3.3 (±0.5 SE) km. All 219 offspring from 49 1^st^ annual broods were sired by the social father as well as all 93 offspring from 20 2^nd^ annual broods when the mother bred with the same male as at the 1^st^ annual brood.

## Discussion

Extra-pair paternity is very low in the barn owl as shown in the present study (six out of 455 nestlings) and another study (one out of 211 nestlings) using an independent sample of broods [19]. Interestingly, six of these seven extra-pair young were found in two 2^nd^ annual broods of females that deserted their first mate (present study) and one young was raised in the 1^st^ annual breeding attempt of the female [19]. This is consistent with the hypothesis that copulations performed while rearing the offspring can allow males to increase their paternity in case their female deserts to start a 2^nd^ annual clutch with another male [18], [27]. From a male point of view, selection may have promoted the evolution of a high copulation frequency to increase the fitness of males who continue to take care of the offspring at the 1^st^ annual breeding attempt while their female produce a 2^nd^ annual brood with another male. From a female point of view, copulating during offspring rearing may be a strategy to convince her male that she will produce the 2^nd^ annual brood with him. This behaviour could induce him to forage intensely not only for the brood but also for his female, who needs extra energy to produce eggs of the 2^nd^ clutch [17]. Another scenario posits that high quality females can afford to desert the 1^st^ brood and are also better to secure extra-pair copulations. This is consistent with the observation that the distance between the two nests of deserting females, for which some offspring at the second nest were sired by the 1^st^ male, was relatively short (less than 1500 m while the distance between nests without multiple paternity was on average 3.3 km). Therefore, multiple paternity may occur only if the distance between the two females' nests is not too large, potentially indicating that females do not store sperm from the 1^st^ male to sire eggs at the second nest. If so, females would have to return to the 1^st^ nest to copulate with the 1^st^ male or, alternatively, the 1^st^ male would have to visit her female at her 2^nd^ nest. An anecdotal personal observation showed that deserting females can indeed continue to visit her 1^st^ nest.

In the barn owl, females that intend to produce a 2^nd^ annual brood benefit from deserting their first brood and breed again with a new mate, since deserting females produce their 2^nd^ annual brood two weeks earlier than non-deserting females [13]. However, the new mates of deserting females are usually yearlings, and hence probably males of low quality, which could explain why deserting females produced a similar number of fledglings as non-deserting females even though they laid significantly more eggs implying that nestling mortality is higher at the 2^nd^ brood of deserting than non-deserting females [13]. As a consequence, deserting females may derive genetic benefits if in the 2^nd^ annual brood some offspring have been sired by the male with whom they produced the 1^st^ annual brood.

The level of multiple paternity that we observed in the barn owl is comparable to the levels observed in other polyandrous bird species where females produce successive clutches with several males who incubate them. In the comb-crested jacana (*Irediparra gallinacea*) only 2.8% of the nestlings were sired by another male than the one who incubated the eggs [28], in the Eurasian dotterel (*Charadrius morinellus*) only 4.6% [29] and in the the red phalarope (*Phalaropus fulicarius*) only 6.5% [30]. This rate is much lower than in some lek-mating species (45% in the wild turkey [*Meleagris gallopavo*] [31]), or in monogamous species (11.1% on average [32]). Because in polyandrous species males invest so much effort in reproductive activities, selection is intense to avoid being cuckolded. This can select for a high copulation frequency (as observed in the barn owl [17], [33], [34]). However, because the second male of deserting females could not prevent their female to copulate with her first male, other behaviour may have evolved to reduce the risk of cuckoldry. For instance, in the wattled jacana (*Jacana jacana*) males remove the first egg of their clutch except at the first annual breeding attempt [35]. This suggests that males from polyandrous species could constrain female extra-pair mating behaviour by imposing high fertility costs to females. If this behaviour also occurs in the barn owl it could explain why we found extra-pair young in only two out of 29 second broods of deserting females. Another possibility to explain the relatively low level of multiple paternity is that the distance between the two successive nests of deserting females was relatively high preventing females to visit their first male.
